# Motor control beyond reach—how humans hit a target with a whip

**DOI:** 10.1098/rsos.220581

**Published:** 2022-10-05

**Authors:** Aleksei Krotov, Marta Russo, Moses Nah, Neville Hogan, Dagmar Sternad

**Affiliations:** ^1^ Department of Bioengineering, Northeastern University, Boston, MA, USA; ^2^ Departments of Biology, Electrical and Computer Engineering, and Physics, Northeastern University, Boston, MA, USA; ^3^ Department of Neurology, Tor Vergata Polyclinic and Laboratory of Neuromotor Physiology, IRCCS Fondazione Santa Lucia, Rome, Italy; ^4^ Department of Mechanical Engineering, Massachusetts Institute of Technology, Cambridge, MA, USA; ^5^ Department of Brain and Cognitive Sciences, Massachusetts Institute of Technology, Cambridge, MA, USA

**Keywords:** human motor control, motor skill learning, control of complex objects, flexible objects, rhythmic movements, discrete movements

## Abstract

Humans are strikingly adept at manipulating complex objects, from tying shoelaces to cracking a bullwhip. These motor skills have highly nonlinear interactive dynamics that defy reduction into parts. Yet, despite advances in data recording and processing, experiments in motor neuroscience still prioritize experimental reduction over realistic complexity. This study embraced the fully unconstrained behaviour of hitting a target with a 1.6-m bullwhip, both in rhythmic and discrete fashion. Adopting an object-centered approach to test the hypothesis that skilled movement simplifies the whip dynamics, the whip's evolution was characterized in relation to performance error and hand speed. Despite widely differing individual strategies, both discrete and rhythmic styles featured a cascade-like unfolding of the whip. Whip extension and orientation at peak hand speed predicted performance error, at least in the rhythmic style, suggesting that humans accomplished the task by setting initial conditions. These insights may inform further studies on human and robot control of complex objects.

## 1. Introduction

The use of whips has a long history, although the modern reader may be mostly familiar with performances in movies or those of whip experts [1]. Trained whip-masters can halve an apple on a person's head without touching their hair or crack two whips in synchrony with music. With its intriguing complexity, cracking a whip showcases the pinnacle of human skill and dexterity. And yet, numerous everyday actions involve the handling of objects that are similarly complex and require a surprising degree of skill, such as tying shoelaces or slipping into a coat. Like the whip, shoelaces or a coat are flexible objects with multiple––in principle infinite––degrees of freedom (DOF) that have significant nonlinearities. While healthy humans handle such apparent complexity with ease, their demands become visible in children and individuals with neurological disorders, where tying shoelaces or putting on a garment can present unsurmountable challenges. Despite the increasing amount of research in motor neuroscience, it remains a mystery how the brain controls and coordinates such intricate interactive behaviors. This study embraces the full complexity of such actions by examining humans handling a whip to hit a target.

In computational motor neuroscience, it is widely agreed upon that the central nervous system relies on internal representations of the body and the environment that are the basis for predicting, planning and executing the appropriate actions [2,3]. One theoretical framework that could account for a range of movement features is stochastic optimal feedback control that plans and generates trajectories based on an internal model of the body optimizing different cost functions [4,5]. However, these studies primarily examined reaching movements, usually confined to two joint degrees of freedom, moving in a horizontal plane, devoid of redundancies or interactions with objects [6]. While such simplifications in the experimental testbeds are necessary for initial advances as they limit confounding variability, some essential aspects of the dynamics are necessarily ‘controlled out’. Little is known about how or whether the control frameworks developed for simple two-dimensional reaching movements scale up to multi-joint movements in three dimensions, let alone to interactions with complex objects, such as a whip [7]. Therefore, this study did not start with specific hypotheses derived from existent control theories, but rather adopted an object-centered or task-dynamic approach to allow insights emerging from the data, specifically from the whip.

The challenges of interaction with non-rigid objects have been highlighted in a considerably smaller set of studies, that were largely conducted in controlled virtual environments to reduce the real-world complexity to physically simpler tasks. For example, several studies on transporting a mass-spring system revealed that human strategies no longer exhibited the minimum-jerk profiles that have been firmly established for unconstrained reaching and that have been replicated by optimal feedback control [8–10]. In previous research, Sternad and colleagues examined the transport of a cup with a rolling ball inside as a proxy for transporting a cup filled with coffee [11–14]. Analyses revealed that typical cost functions, such as minimizing expended force, were subordinate to other priorities such as stability and predictability of the interaction [15,16]. Other work on bouncing a ball with a paddle [17], juggling [18] or balancing an inverted pendulum [19–21] highlighted how human control strategies are governed by the object's dynamics.

Importantly, these studies anchored their inquiry in the analysis of the objects that were moved, such as the physics of the racket-ball impact or of the cup and ball interactions. As this task-dynamic approach examined the dynamics of the object first, it is free of any *a priori* assumptions about control [22]. Assuming a given task goal, i.e. transporting the cup without losing the ball, mathematical analysis of the object's dynamics then revealed the solutions that achieved this goal. As the task inevitably created a high degree of redundancy, there existed a mathematically infinite number of solutions, i.e. a solution manifold. Which solution in this subset was actually employed by humans could be probed by comparing different control objectives, such as stability or predictability. To extend this strictly model-based research to real-world tasks and embrace their full realistic complexity, this study aimed to investigate how humans handle a whip. Specifically, we examined how humans handle a 1.6-m-long bull whip to hit a target.

Following the task-dynamic approach from our previous work [22], this study addressed this rich behaviour by analysing the whip and its interaction with the hand. Agnostic about the specific controller, we first examined the whip's pattern evolving towards the target. We hypothesized that successful handling of the whip was characterized by smooth and ‘simple’ patterns from the throw onset to hitting the target. We conjectured that a simple and smooth evolution of the whip made the infinitely-dimensional object predictable and therefore manageable. As the evolution of this complex object depended on the hand movements, we analysed the kinematics of the hand to identify the input that generated such whip patterns.

Note that the hand–body system itself is also highly redundant, and a large variety of multi-joint patterns had to be expected. To tease apart what was essential to manipulating the whip in a target-oriented fashion, participants were asked to hit a target using two different styles: as single discrete actions separated by pauses, and in continuous rhythmic fashion. Discrete actions that feature a clear start and end and rhythmic actions that are repetitive and periodic have been explored in separate lines of research [23–25]. To extract the essence of hitting a target with a whip, this study tested participants performing the same task in both styles to identify similar elements and differences.

## 2. Methods

### 2.1. Participants

A total of 16 healthy adults (7 males, 9 females, average age 26.6 ± 5.0 years) volunteered for the experiment. Prior to participation, they read and signed an informed consent form for the protocol which was approved by the Institutional Review Board of Northeastern University. Fifteen participants reported being right-handed and one ambidextrous; none had any prior neurological or musculoskeletal disorder. Four male participants reported moderate prior experience with cracking a bullwhip, all others reported none. The variety of skill levels helped to reveal the variety of strategies and the invariant features employed in this new task.

### 2.2. Experimental task and data acquisition

**Figure 1.**
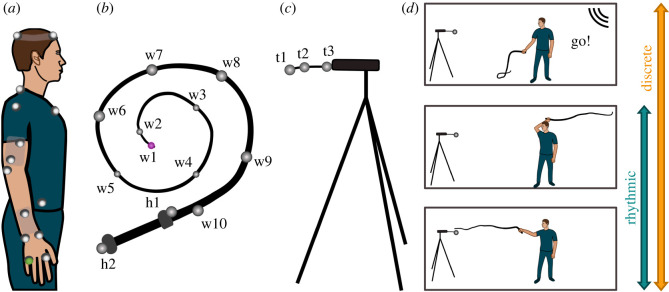
Experimental setup and protocol. (*a*) Placement of markers for three-dimensional motion capture. Four markers were placed on the head, four markers on the torso, two markers on the hips, eight markers on the right arm. Two markers on the upper arm and one marker on the lower arm were fixed on stiff rubber bases; the marker at the metacarpal head V (highlighted in green) was used to quantify hand movements. (*b*) Ten soft and light custom-developed markers were secured on the thong of the whip (flexible and tapered part, 1.6 m long), two customized soft markers were placed on the handle of the whip (rigid proximal part, 0.24 m long); the marker on the tip is highlighted in pink. (*c*) Three customized soft markers were located on the target, made of a metal spring, connected via a plastic rod to a bar that was mounted on a tripod. The markers on the whip and on the target are displayed in real scale relative to the whip and the target, markers on the body had diameters of 1.2 cm and are enlarged for illustration. (*d*) Experiment layout. The target faced participants, its height matched the participant's shoulder height, the distance to the participant was adjusted to provide a 5-cm overlap with the whip when both the whip and the right arm were fully extended.

Participants were instructed to hit a target with a 1.6-m-long bullwhip. Kinematic data were recorded using 12 Oqus 3 + cameras at a sampling frequency of 500 Hz (Qualisys, Goetheborg, Sweden). Participants wore tight dark-coloured clothes without reflective elements and were provided with transparent plastic glasses to protect their eyes. Eighteen reflective markers of diameter 1.2 cm were placed on the participant's body (figure 1*a*): four on the head (anterior and posterior, left and right), four on the torso (left and right acromia, 7th cervical vertebrae, sternum jugular notch), two on the hips (ilia anterior superior), six on the right arm (lateral epicondyle of humerus, wrist at the styloid processes of ulna and radius, a rubber-base with two markers on the upper arm and a similar base with one marker on the lower arm) and two on the hand (metacarpal heads II and V). This study only analysed the kinematics of the hand, using the metacarpal head V marker.

A commercially available bullwhip was used for the experiment (Ardour Crafts, USA). The original whip consisted of four parts: the rigid plastic handle covered with braided leather, the thong, made of layered braided leather tapering toward its distal end, the fall, a thin single band of leather and the cracker, a thin string. Fall and cracker were removed for the experiment to facilitate the recording and reduce air-drag and turbulence effects. The remaining thong measured 1.60 m and the rigid handle measured 0.24 m; their total mass was 336 g. We will refer to the thong as the ‘whip’ in this study.

Tracking of the whip's motion with conventional markers proved challenging as they easily detached from the whip due to its speed and contacts with the floor and target [26]. Therefore, we custom-designed markers from light-weight sponge balls (soft poly-foam, diameter 2 cm, mass 0.3 g, w1 to w5). They were large enough to be detected by the cameras during the fast motion and soft enough to resist and dampen impacts while remaining sufficiently light-weight to not compromise its dynamics; they were covered with reflective tape. The markers at the thicker part of the whip (w10 to w6) were slightly larger sponge balls (diameter 5 cm, mass 1.3 g). The distance between adjacent markers (w10 to w3) was 19 cm, except the two most distal markers w1 and w2 that were at 6 cm separation. These 10 markers were threaded into the thong and two additional markers were placed onto the handle (h1, h2; figure 1*b*).

The target consisted of a horizontal bar extended by a horizontal spring mounted on an approximately 1.5 m-high tripod that was loaded with a 5 kg weight to ensure its stability (figure 1*c*). A customized marker t1 (soft poly-foam with metal core, diameter 5 cm, mass 9 g) was attached to the free end of the horizontal spring. Two markers t2 and t3 (diameter 5 cm, mass 1.3 g) were affixed to the spring and the rod to increase the visibility of the target.

### 2.3. Experimental procedure

Participants were positioned at approximately 2.2 m from the target with one foot standing on a line taped on the floor; they could place the other foot comfortably behind it, farther from the target (figure 1*d*). The distance to the target was adjusted for each participant such that the fully extended arm plus whip had a 5 cm overlap with the target. The height of the target was also adjusted to match participants' shoulder height.

**Figure 2.**
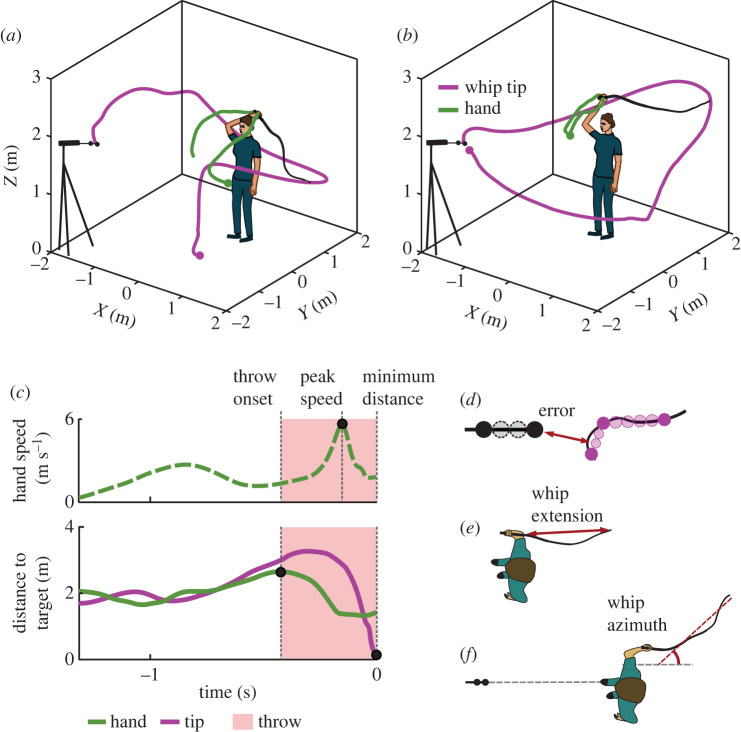
Exemplary trajectories and metrics definition. (*a*) Exemplary trajectories of the hand and the tip of the whip in discrete style. A discrete trial started with the whip on the ground and ended when the tip of the whip reached its minimum distance to the target. (*b*) Rhythmic style. A rhythmic trial was defined as lasting between two instances of whip-to-target minimum distance. The hand path is green, the path of the tip of the whip is magenta. The dots denote the beginning of the trial. The position of the participant and the whip are at the moment of maximum distance between the hand and the target that defined the onset of the throw. (*c*) Exemplary time profiles of one discrete trial showing hand speed (dashed green), tip-to-target distance (solid magenta) and hand-to-target distance (solid green). Time is displayed backwards from minimum distance (throw interval). The circles denote three temporal landmarks of the throwing action used to segment each trial. The colour shading highlights the throw interval between throw onset and minimum distance. (*d*) The error was defined as the minimum distance between the whip and the target. The minimum distance was obtained via interpolating the whip in between three distal markers (dark magenta), interpolating the target between the two distal markers (black markers), and finding the minimum among the pairwise distances of the interpolated values (an exemplary minimum pair is shown with arrow). (*e*) Whip extension. At peak speed of each trial, the Euclidean distance between markers w1 and w10 was found. That distance was divided by the whip's arclength to define the whip extension. (*f*) Whip azimuth. At peak speed of each trial, the whip was fitted with a line in the *xy*-plane to determine the direction at its largest extent. Whip azimuth was defined as the angle of that line, with respect to the target direction.

Participants were instructed to use the bullwhip to hit the target in discrete and rhythmic fashion. The experimenter first demonstrated the task using a modification of the ‘overhand crack’ [1]: the whip was cast underarm backward, then thrown forward in an overarm fashion. In the discrete style, participants started with the whip resting on the floor in front of them (figures 1*d* and 2*a*). At the ‘Go!’ cue, they attempted to hit the target; the whip was then returned to the starting position. In the rhythmic style, participants performed continuous movements in a self-paced manner, without stopping the movement or contacting the floor with the whip (figure 2*b,* see also videos in Supplementary Material).

For each style, five consecutive blocks were recorded. For the discrete style, the total duration of one block was 180 s. This allowed 33 ± 3 whip throws (trials) per block. For the rhythmic style, each block was 40 s long to allow for the same approximate number of cycles (trials) per block. Participants took breaks for 1–2 min between blocks. The number of trials was determined after preliminary assessments of the physical exertion needed especially in the rhythmic version of the task; the goal was to maximize the number of repetitions without causing fatigue. Nine participants performed the discrete style first, seven performed the rhythmic style first.

### 2.4. Data processing

All marker data were low-pass zero-lag filtered with a finite impulse response (FIR) filter. The low-frequency cutoff was set to 20 Hz for the body markers and 40 Hz for the whip markers, following inspection of the residuals. Passband and stopband frequencies were 1 Hz below and above the cutoff frequency; the stopband attenuation was −60 dB, the passband ripple was 0.1. The design method used Kaiser windowing, and the minimum filter order was determined automatically via a function *designfilt* with *lowpassfir* argument. The position signals were afterwards time-differentiated to obtain first- and second-order derivatives. All analyses were carried out with custom-written software and built-in functions in Matlab (Mathworks, Natick, MA). For more details on the recording and preprocessing of the data see [27].

### 2.5. Landmarks in whip and hand movements

To parse the continuously recorded blocks into individual trials, the minimum distance between the target marker and the whip defined the end of a trial in both styles. In the discrete style, the beginning was defined when the hand speed exceeded a 0.5 m s^−1^ threshold just before the vertical coordinates of the three most distal whip markers exceeded 2 cm, i.e. detecting the lift of the whip from the floor. In the rhythmic style, the start of each trial coincided with the end of the previous one.

To define more task-specific metrics, the continuous movements of the whip and hand were parsed into functional segments. Using the marker on the hand (V-th metacarpal head), the three distal whip markers, w10, w9 and w8, and the target markers, t1, t2 and t3, three temporal landmarks were defined for each trial: (1) *throw onset*, defined as the time at which the hand was pointing backward at maximum distance from the target; (2) *peak speed*, defined as the instant when the hand's tangential velocity (speed) reached its maximum; (3) *minimum distance*, defined as the instant when the whip was at minimum distance to the target (see details in the section below). Reflecting our emphasis on the late trial features, zero time was set at minimum distance and the time axis is shown backwards in the figures. Figure 2*c* illustrates these definitions by showing the time series across the entire *trial interval* with hand speed (green dashed), the instantaneous distance between the hand and the target (green), and the instantaneous distance between the whip and the target (pink). The three temporal landmarks are marked by vertical lines. The shaded interval denotes the *throw interval*.

### 2.6. Hitting success and error

Two metrics quantified the participants' task performance: *success rate* and *error*. *Success rate* was defined as the percentage of trials within a block that resulted in target hits. To determine those, oscillations of the target caused by contact with the whip were used as indicators. In the rhythmic style, it occasionally happened that the target was hit while still oscillating from a previous hit; such cases were evaluated manually. A more fine-grained metric to quantify performance was the error, defined as the minimum distance between the whip and the target. To obtain the *minimum distance,* the whip between the three distal markers and the target between the two distal markers were interpolated; the minimum distance was determined among the pairwise distances of the interpolated values over the time of the whip throw (figure 2*d*).

### 2.7. Rhythmicity

To assess whether participants indeed performed the rhythmic style in a regular rhythm, the temporal variability was quantified. For comparison, temporal regularity was also quantified for the discrete style. To this end, the *inter-trial interval* was defined as the time between the start of two consecutive trials. This metric coincided with the trial interval in the rhythmic style. For the discrete style, it included additional events, such as slowing down the whip, returning it to starting position and pausing before the next ‘Go’ cue. The metric *rhythmicity* was defined as the coefficient of variation (CoV) of the inter-trial interval within one block of trials.

### 2.8. Whip speed profiles

The time series of tangential velocity of all 10 markers on the whip were computed to examine the evolution of whip motion during the throwing action. To obtain a mean profile for each marker, the speed profiles from throw onset to the time of minimum distance were time-normalized to the mean duration of that interval (see the shaded area in figure 2*c*); then the average and standard deviations were obtained for each time point. A mean profile was calculated for each participant, separately for the discrete and rhythmic styles. Zero time was set at a minimum distance and time was normalized to dimensionless units. The *peak tip speed* of the whip marker w2 was determined (w1 marker was too noisy to be differentiated and w2 was only 6 cm adjacent to it).

### 2.9. Whip extension and orientation at peak hand speed

To evaluate the whip shape, two metrics were defined at the peak hand speed landmark. *Whip extension* was quantified as the Euclidean distance between the distal handle marker (h1) and the tip of the whip (w1), as illustrated in figure 2*e* (total length of the whip: 1.6 m). The whip's orientation in the horizontal plane, i.e. *whip azimuth*, was estimated by fitting a line to the whip and handle markers (w1 through w10 and h1) using singular value decomposition (the whip was nearly straight at peak hand speed). *Whip azimuth* was defined by the angle between the horizontal projection of the direction vector of that line with respect to the target direction (figure 2*f*).

### 2.10. Hand kinematics

As for the whip speed profiles, the temporal evolution of the tangential velocity of the hand (using the V-th metacarpal head marker) was examined, although on a longer interval that spanned the full trial. To obtain a mean profile for each participant in each style, the hand speed profiles were time-normalized to the mean trial duration of the selected trials, i.e. from the trial start to the time of minimum distance, and then were averaged. With few exceptions, the profiles of the hand speeds in both styles displayed two peaks. The first slower peak corresponded to lifting the whip from the floor to cast it backward in the discrete style; in the rhythmic style it included the follow-through from the previous cycle. While these were functionally very different phases of the movement, the second and invariably higher peak marked the crucial moment of the whip throw. Therefore, *peak hand speed* was determined for each trial as the maximum value of the speed profile. Zero time was set at the moment of minimum distance.

In order to evaluate the orientation of the hand holding the whip handle, markers on the hand and on the handle were used to define a rigid body. The axes were defined by the average position of w10 and h1, position of h2, and the average position of the two hand markers. The orientation of this hand-handle was defined by the angle of its horizontal projection in relation to the sagittal line extending from the target to the body.

### 2.11. Statistical analyses

Prior to preprocessing and analysis, the data were scanned and 49 trials out of a total of 5276 trials had to be discarded from all analyses. The two main exclusion criteria were that more than five markers had simultaneous gaps or that participants had problems in executing the task. The discarded trials were distributed across 11 of 16 participants, hence the elimination did not introduce any bias*.* For normalizing and averaging the speed profiles of the hand and the whip, 193 more trials (3.9%) were discarded due to having longer than 200-frame (0.4s) gaps; those eliminated trials were distributed across 10 participants.

In reporting the summary results, means and standard deviations were computed for all metrics, except error and whip extension that exhibited skewed distributions; median and inter-quartile ranges were reported for those two.

For the analysis of success rate, a binary response variable (hit or miss), the data were fitted with a binomial distribution with a *logit* link function and evaluated with a generalized linear mixed model (GLMM). For all other metrics, the dependent measures were modelled as a linear combination of fixed and random effects and evaluated by a linear mixed model (LMM).

The main experimental design compared discrete with rhythmic style across five blocks , i.e. it was a 2 × 5 mixed-effect model. This mixed model compared the experimental conditions (fixed effects: style and block), while accounting for the variability between participants (random effect: participants). To identify the model that best fit each variable, an iterative procedure was adopted to assess whether the inclusion of main effect, interaction and random effects was justified [28–30].

The model that we considered to fit all variables, including all the possible factors, was the following:

2.1 $$Y_{ij}=(\beta_{0}+P_{0i})+(\beta_{S}+P_{Si})S_{j}+(\beta_{B}+P_{Bi})B_{j}+\beta_{SB}S_{j}B_{j}+\epsilon_{ij}\;,$$

where *Y* was the response variable or dependent measure for each participant *i* and each trial *j*, *S* was the style condition (two levels: discrete and rhythmic), B was the block number (from 1 to 5), *β* were the fixed-effects coefficients, *P* were the random-effects coefficients for participants and $\epsilon_{ij}$ were the residuals. The interaction term SB tested whether the slope of one factor, i.e. block number B, was significantly different from the slope of the interacting factor, i.e. style S. A positive slope for style would mean that the variable was larger in the rhythmic style, a positive slope for block would mean that the variable increased from block 1 to block 5 in the discrete style; a zero slope for the style × block interaction would mean the same block effect in the rhythmic style as in the discrete style, while a positive slope would mean an increased block effect in the rhythmic style, compared to the discrete style.

To assess the possible contribution of the hand's and whip's initial conditions to the resulting error, correlations with error were calculated for the whip extension, whip azimuth, peak hand speed and peak tip speed. These correlations were obtained through the following linear mixed model (LMM):

$$E_{ij}=(\beta_{0}+P_{0i})+(\beta_{S}+P_{Si})S_{j}+(\beta_{X}+P_{Xi})X_{j}+\beta_{SX}S_{j}X_{j}+\epsilon_{ij},$$

where *E* is error, *X* is a variable of interest, and the other terms are defined above. This model tested whether the variable of interest correlated with error in each style while accounting for inter-participant differences. Correlation of the two latter parameters (peak hand speed and peak tip speed) was also assessed in a similar manner.

Statistical analyses were carried out in Matlab (Mathworks, Natick MA), with functions *fitlme*, *fitglme* and *lratiotest* from the Statistics Toolbox.

## 3. Results

Participants were instructed to hit a target using a hand-held bullwhip comprising a rigid handle and a flexible tapered thong (figure 1). Each participant performed the task in a discrete style with pauses between individual attempts, and a rhythmic style with individual trials connected into a continuous sequence (figure 2*a,b*, see electronic supplementary material). The performance of 16 participants was evaluated by success rate, error and rhythmicity in the two styles, and improvements were assessed across the five blocks of trials. The central interest was in the whip behaviour and how its initial conditions at peak hand speed resulted in its unfolding towards the target. Characteristic features of the hand trajectories were correlated with whip variables to explore how hand trajectories related to task success. The comparison between discrete and rhythmic styles revealed differences and patterns that were essential to throwing a whip to hit a target.

### 3.1. Task performance

**Figure 3.**
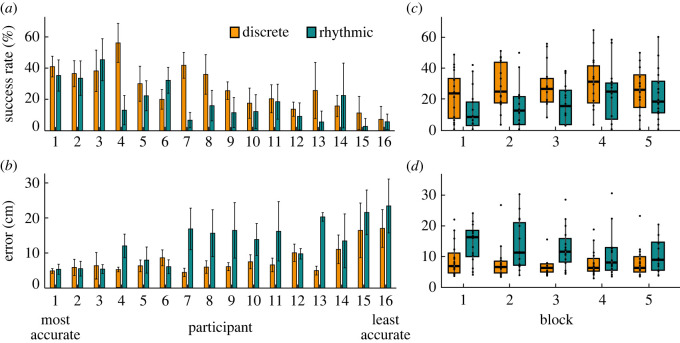
Performance metrics across participants and across blocks. (*a*) Success rate. Each bar represents the percentage of successful trials in each participant separated by style. (*b*) Error calculated as minimum distance between the final segment of the whip and the target. Median error for each participant separated by style. Participants are ranked and numbered based on the median error across both discrete and rhythmic styles. All five blocks were pooled together and averaged for each style and participant. The error bars show standard deviations within five block values. (*c*) Success rate. (*d*) Error. The data of all participants were pooled. Performance metrics are aggregated within each block and style. Horizontal bars in every box depict the median, the box dimensions depict the interquartile range (IQR), and the error bars indicate the 1.5-IQR-deviation from the median; points represent the individual values of the participants.

All 16 participants succeeded in hitting the target multiple times in both styles. The success rates and errors for each participant in both styles across all blocks are shown in figure 3*a,b*. The success rates exhibited a large range from 0% to 71% per block per participant and the errors similarly varied from a block median of 3 cm to 35 cm per block. For better visualization, participants were rank-ordered and numbered according to their median errors calculated across both styles. Visual inspection shows that the discrete style tended to exhibit higher success rates and lower errors in most participants.

Aggregating these variables into blocks enhanced this observation. Figure 3*c* and *d* show that the success rates were significantly higher in the discrete style, and errors were always higher in the rhythmic style (main effect of style for success rate, *β* = −1.11, *p <* 0.001; main effect for error *β* = 0.10, *p <* 0.001). In the rhythmic style, both metrics showed significant improvements across blocks (respectively, *β* = 0.15, *p <* 0.01; *β* = −0.009, *p <* 0.001). By contrast, in the discrete style, the performance metrics did not show any improvements (respectively, *β* = 0.05, *p =* 0.327; *β* = −0.003, *p =* 0.382). Note that while the rhythmic style appeared more tiring as the whip had to be kept in the air for the entire block, the improvements indicated that fatigue seemed to only play a minimal role for task success.

**Figure 4.**
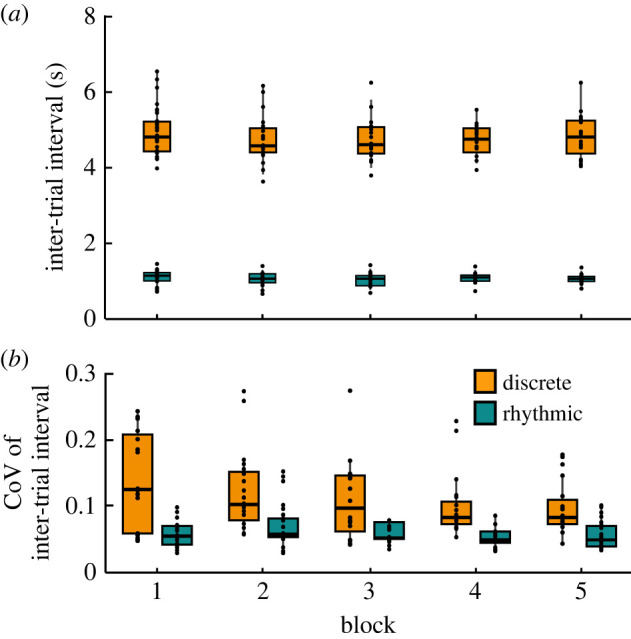
Timing and rhythmicity in blocks and styles: (*a*) Inter-trial interval. (*b*) Coefficient of variation of inter-trial interval. The data of all participants are pooled. The metrics are aggregated within each block and style. Horizontal bars in every box depict the median, the box dimensions depict the interquartile range (IQR), and the error bars extend to the farthest point within 1.5-IQR-deviation from the median; points represent the individual values of the participants.

For the evaluation of rhythmicity of the task execution, specifically in the rhythmic style, the trial durations were calculated and their variability was quantified by the coefficient of variation. As expected from the instruction, the inter-trial intervals were longer in the discrete compared to the rhythmic style (*β* = −4.16, *p <* 0.001; figure 4*a*). Inter-trial intervals decreased slightly across blocks in the discrete style (*β* = −0.05, *p* < 0.01), but not in the rhythmic style, as indicated by the interaction effect (*β* = 0.04, *p <* 0.01).

Not surprisingly, the variability was smaller in the rhythmic style (*β* = −0.112, *p <* 0.001), with a mean of 0.06 (s.d. 0.03) compared to 0.12 (s.d. 0.08) in the discrete style (figure 4*b*). This number signified a high degree of periodicity in the rhythmic performance that did not change across blocks (*β* = 0.015, *p* < 0.05, significant interaction). In addition, the values in the discrete style were also surprisingly low, suggesting a modest degree of rhythmicity that evolved over the experimenter-participant dyad. This variability even decreased across blocks (*β* = −0.017, *p* < 0.001).

### 3.2. Whip kinematics

**Figure 5.**
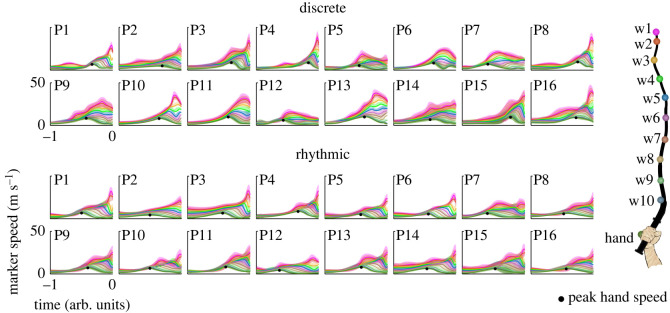
Whip markers speed. Speed profiles of the hand and whip markers during the throw interval of all participants in discrete and rhythmic styles. The tangential velocities (speeds) of each marker are shown with lines with colour coding depicted at the right. Shading depicts one standard deviation of speed for each marker. Peak hand speed is highlighted with black circles. The speed data were acquired from all trials of each style and participant on the interval from throw onset to the minimum distance (see red shaded area in figure 2*c*); they were aligned at the moment of minimum distance (time 0), time-normalized and averaged. Participants are ranked by ascending median error in both styles as in figure 3.

The evolution of the whip was characterized by the speed profiles of the 10 whip markers from the handle to the tip of the whip. Figure 5 displays the mean tangential velocities (speeds) of each marker from throw onset to minimum distance, i.e. across the throw interval, for both styles (see methods for definition of throw onset and minimum distance). As in figure 3, participant numbers reflect their rank order defined by the median errors across both styles (P1 had the smallest errors, P16 had the largest errors). To obtain the mean speed profiles, the time was normalized to the mean throw interval in each participant and style. Figure 5 shows that while participants displayed different whip speed profiles, the more proximal markers tended to reach peak speed first with the adjacent more distal markers following in sequence. This pattern suggests an unfolding of the whip towards the target with energy propagated along the whip. With few exceptions, the tip of the whip reached its peak speed close to the minimum distance. Some deviations from this segment-wise unfolding are likely due to waves reflected back to the handle or other uncontrolled movements of the whip (see videos in Supplementary material). Despite the overall pattern, individual participant variations did not show visible correlations with their task performance.

**Figure 6.**
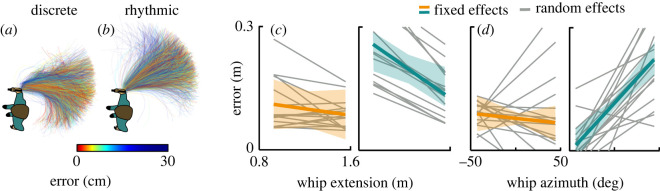
Whip variables. Whip extension and whip azimuth at peak hand speed and their correlations with error. (*a, b*) Top-down view of the whip configurations at peak hand speed in the discrete and rhythmic styles. Each panel depicts all trials of all participants in one style at the same landmark. The target is on the left and is not shown. The colour gradients of the whip depict the error in each trial. All whip snapshots were translated in space to have the same position of the handle. The same participant's posture is shown for illustration. (*c, d*) Correlations of whip extension and whip azimuth at peak hand speed with error (minimum distance between whip and target, figure 2*d*). The slopes for the two styles were obtained from the linear mixed model. The coloured lines and shading depict the fixed-effect estimates and standard deviations; the grey slopes for each participant and style constitute random plus fixed effects.

Therefore, two whip variables, whip azimuth and whip extension, were investigated at the landmark of peak hand speed. Even though the hand trajectories were extended in time, the moment of peak hand speed can be regarded as critical for the throw and its target accuracy because after that moment the hand decelerated. The whip's shape with its extension and azimuth are displayed in figure 6*a,b* for all trials in both styles; the colour corresponded to performance error in each trial. The 1.6-m long whip was extended to a median of 1.45 (IQR 0.14) m in the discrete style throughout all blocks (block effect *β* = 0.002, *p =* 0.513); in the rhythmic style it was extended to 1.43 (IQR 0.07) m (style effect *β* = 0.037, *p* = 0.337) and it also increased with practice (interaction effect *β* = 0.006, *p* < 0.05), indicating a contribution to performance improvement in the rhythmic style. The whip azimuth was −10.9 deg (s.d. 21.5 deg) in the discrete style, indicating a relatively straight backward orientation. This angle was significantly larger in the rhythmic style with a mean of 28.4 deg (s.d. 16.1 deg; *β* = 39.43, *p* < 0.001), suggesting that the whip was pointing mostly to the right as shown in figure 6*a,b*. That orientation remained unchanged throughout all blocks (block effect *β* = −0.230, *p* = 0.76; no interaction).

To examine to what degree these initial whip variables determined the task performance, both extension and azimuth were correlated with hitting error. While whip extension showed no correlation in the discrete style (*β* = −0.040, *p* = 0.163), this correlation was strongly negative in the rhythmic style (*β* = −0.153, *p* < 0.001, figure 6*c*). These results suggested that in the rhythmic style, straightening the whip at peak hand speed reduced the error. While the whip azimuth did not show a significant correlation with error in the discrete style (*β* = −0.0002, *p* = 0.54), it was positively correlated in the rhythmic style (*β* = 0.0026, *p* < 0.001), as illustrated in figure 6*d*. This highly significant correlation suggests that pointing the whip straight behind oneself at that landmark facilitated accuracy in the rhythmic style.

**Figure 7.**
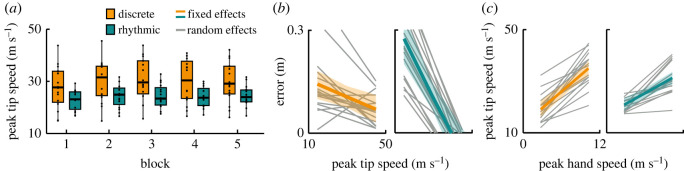
Whip tip speed. (*a*) Peak speed of the tip of the whip. The data of all participants were pooled. The values are aggregated within each block and style. Horizontal bars in every box depict the median, the box dimensions depict the interquartile range (IQR) and the error bars indicate the 1.5-IQR-deviation from the median; points represent the individual values of the participants. (*b*) Correlation of peak speed of the tip of the whip with error (minimum distance between whip and target, figure 2*d*). The slopes were acquired by means of linear mixed model, displayed separately for the discrete and rhythmic styles. The coloured slopes and the confidence bands depict fixed effect estimates and standard deviations; slopes for each participant and style are displayed in grey and constitute random plus fixed effects. (*c*) Correlation of peak hand speed with peak tip speed. The correlation slopes were acquired by means of linear mixed model, displayed separately for the discrete and rhythmic styles. The coloured slopes and the confidence bands depict fixed effect estimates and standard deviations; slopes for each participant and style are displayed in grey and constitute random plus fixed effects.

Together with orientation and extension of the whip, the speed of its tip was evaluated and displayed in figure 7*a*. Peak tip speed was on average 29.7 (s.d. 7.8) m s^−1^ in the discrete style and was significantly different from the slower speed in the rhythmic style: 24.1 (s.d. 3.6) m s^−1^ (*β* = −5.02, *p* < 0.01). It slightly increased with blocks in the discrete style (*β* = 0.448, *p* < 0.05), but not in the rhythmic style, as indicated by a significant interaction (*β* = −0.215, *p* < 0.001). Notably, peak tip speed correlated with error in both styles (discrete: *β* = −0.002, *p* < 0.05; rhythmic: *β* = −0.008, *p* < 0.001) as illustrated in figure 7*b*.

How did the hand generate these patterns of the whip motion? To investigate how whip behaviour was related to hand behaviour, peak hand speed was correlated to the speed of the tip of the whip. Figure 7*c* shows a significant correlation in both styles (hand peak speed main effect: *β* = 3.33, *p <* 0.001; interaction with style: *β* = −1.11, *p <* 0.001) indicating that for both styles higher peak hand speeds generated higher whip peak speeds.

### 3.3. Hand kinematics

**Figure 8.**
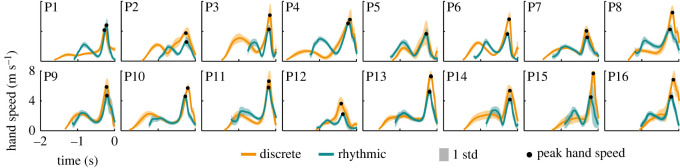
Profiles of hand speed (tangential velocity). The profiles in both styles were aggregated across trials within each style and participant, time-normalized to the entire trial interval, averaged and aligned at the time of minimum distance (time set to zero). The solid lines represent the mean trajectory, the shaded bands one standard deviation around the mean. Black circles denote peak hand speed as in figures 2 and 5. Participants are ranked by ascending median error in both styles as in figure 3.

Profiles of the tangential velocity of the hand marker h1 are shown in figure 8. Each panel displays the mean speed profiles in both styles for each participant (in the same rank order as above). Time was normalized to afford averaging, and subsequently rescaling by its respective mean trial interval. The speed profiles were aligned to the time of minimum distance of the tip to the target. The profiles for the two styles were remarkably similar within each participant, seen by the relatively narrow standard deviation bands. All participants, except P1 and P15, featured two distinct peaks, suggesting two phases of the movement. However, the first peak pertained to very different actions in the two styles: in the discrete style, the first phase was associated with lifting the whip from the floor and casting it backward; in the rhythmic style that first phase included the follow-through from the previous cycle and casting the whip for the new throw. By contrast, the second and invariably higher peak corresponded to the same critical moment in both styles when the hand was accelerated to reach its peak. During the subsequent deceleration interval, the whip unfolded as the change in acceleration injected energy into the whip.

**Figure 9.**
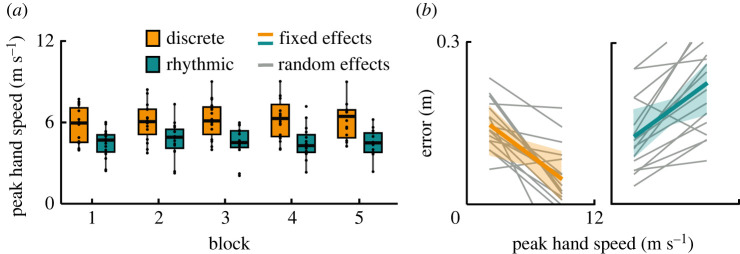
Peak hand speed. (*a*) The data of all participants were pooled. The values were aggregated within each block and style. Horizontal bars in every box depict the median, the box dimensions depict the interquartile range (IQR), and the error bars indicate the 1.5-IQR-deviation from the median; points represent the individual values of the participants. (*b*) Correlation of peak hand speed with error (minimum distance between whip and target, figure 2*d*).

For more detailed comparisons of the hand speed, the peaks of the profiles were examined. The peak hand speed was 6.1 (s.d. 1.3) m s^−1^ in the discrete style and only 4.5 (s.d. 1.0) m s^−1^ in the rhythmic style, which was a significant difference (*β* = −1.24, *p <* 0.001). In the discrete style, the peak speed increased slightly across blocks (*β* = 0.09, *p <* 0.05), but not in the rhythmic style, as shown by a significant interaction (*β* = −0.11, *p <* 0.001; figure 9*a*). Further exploration of the peak hand speed in their relation to error revealed a striking difference between the two styles (figure 9*b*): in the discrete style, faster hand movements were associated with smaller errors (*β* = −0.015, *p* < 0.01), while the positive slope in the rhythmic style indicated the inverse relation: higher peak speeds were associated with larger errors (*β* = 0.029, *p* < 0.001).

**Figure 10.**
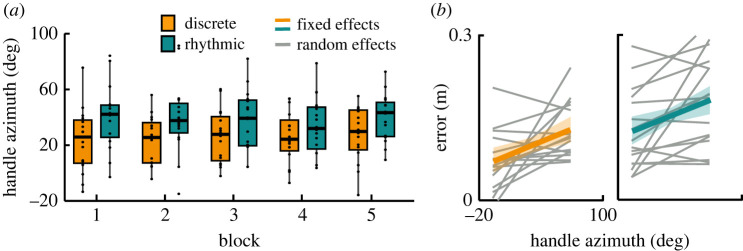
Hand azimuth. (*a*) Hand azimuth at peak hand speed for each block and each style pooling data of all participants. Horizontal bars in every box depict the median, the box dimensions depict the interquartile range (IQR), and the error bars indicate the 1.5-IQR-deviation from the median; points represent the individual values of the participants. (*b*) Correlation of hand azimuth with error for both styles. Each line indicates one participant. Error was calculated as the minimum distance between whip and target (figure 2*d*).

In addition, the orientation of the hand at peak hand speed was examined, similar to the whip variables. Figure 10 displays the azimuth angles of the hand-handle across blocks and styles. The average azimuth in the discrete style was 24.3 deg (s.d. 18.0 deg) and 36.1 deg (s.d. 18.8 deg) in the rhythmic style; the latter was significantly larger (*β* = 16.6, *p <* 0.001). While the angle showed a small but significant increase across blocks in the discrete style (*β* = 1.4, *p* < 0.05), it did not change in the rhythmic style (interaction *β* = −1.6, *p* < 0.01). The hand azimuth correlated positively with error in both styles (*β* = 0.0007, *p* < 0.05). Correlating hand azimuth with whip azimuth rendered significant positive correlation in both styles, despite differences in the mean values of the two variables (*β* = 0.208, *p* < 0.001).

## 4. Discussion

This study examined how humans manipulated a highly nonlinear flexible object with infinite dimensions––a whip. Counter to scientific convention to isolate the features of interest, either by experimental reduction or by modelling the task in a virtual environment, this study embraced the full complexity of natural behaviour. This is grounded in the understanding that such a complex action is not the sum of its parts but needs to be examined in its entirety. One first problem of this approach is that, when allowing unconstrained movements interacting with the whip, a wide variety of individual behaviors emerged. This is not only due to participants' different degrees of coordinative abilities, but also due to the redundancy of the task that allows an infinite variety of solutions, even when perfect. Nevertheless, regularities emerged from this richness of behaviour that allowed a number of insights into how humans control the 1.6-m-long flexible object to hit a target.

### 4.1. An object-centered or task-dynamic approach

To disentangle this highly complex dataset, this study followed an object-centered approach. Instead of probing into the multi-joint kinematics of the human subjects to directly address questions on control priorities, the analysis started by turning its attention to the whip for insight into what mattered for successful task completion. This analysis strategy followed our previously developed task-dynamic approach that begins with the analysis of the object's physics to identify variables and their combinations that achieve task success under given objectives [15,16,22,31]. Only in a second step, the data from human subjects are related to the physical-mathematical analysis of the manifold of solutions afforded by the task. However, this previous research examined complex objects that were mathematically modelled as the experiments were conducted in a virtual environment. Here, we aimed to extend this approach to unconstrained real-world behaviour where a full mathematical model of the object was not (yet) available. In this spirit, we started with the hypothesis that good performance was indicated by ‘simple’ patterns of whip motion. Reduced complexity of the whip would reflect control of the whip to achieve the task goal, hitting the target. Simplicity of the whip pattern was examined by the whip's unfolding and its initial conditions. Note that simplifying the dynamics of the object also increases the predictability of interaction, thus facilitating control [13,16,32].

### 4.2. Initial conditions to simplify the whip's behaviour

As an analytical model of the whip was not available for this full three-dimensional real behaviour, the whip's dynamics were characterized by a set of novel metrics that were then related to task achievement, i.e. error. The degree of simplification of the high-dimensional nonlinear whip-hand system was first examined by the temporal evolution of the 10 whip markers. Extracting the whip markers' speed profiles toward the target revealed a cascade of adjacent markers from handle to the tip reaching peak speed just prior to reaching the target. Higher whip tip speed correlated with higher accuracy in hitting the target in both styles, suggesting that a faster whip movement might be more successful, counter to the classical speed-accuracy trade-off literature [33,34]. Also as one may expect, the faster the whip, the faster the hand's peak speed. However, peak hand speed displayed a correlation with error similar to the whip's tip speed, but only in the rhythmic style.

Two metrics that compressed the high-dimensional whip were defined: whip orientation and whip extension at peak hand speed. The rationale was that simplification of the whip–hand system might be achieved by controlling initial conditions that determine the whip's evolution [35,36]. Even though the time mark of peak hand speed was embedded in the continuous motion, the following cascade of peak speeds in the whip markers suggest that indeed peak hand speed acted as a key landmark. The whip configurations revealed that for best performance in the rhythmic style, the whip was fully extended with an azimuth angle of 28.6 deg (s.d. 4.5 deg). In the discrete style, those regularities were absent, in lieu of a clear correlation of accurate target hits with peak speed of the tip of the whip. The fact that these scalar metrics seemed to inform about performance suggests that the human actor organizes the complex high-dimensional dynamics into simpler subsystems.

At peak hand speed also the hand and the handle were oriented backwards, correlating positively with the whip azimuth. Such configuration led to better performance, especially in the rhythmic style. Therefore, one might speculate that to better control the whip, participants pointed the handle backward to orient the whip backward while extending it maximally to render it into a rod-like object. Taken together, these trends support the hypothesis that humans simplified the interactive dynamics by exciting the simple modes of the whip dynamics to successfully achieve the task. This interpretation is consistent with first advances on modelling whip patterns supporting that the whip takes on relatively simple shapes during human manipulation [37]. Nevertheless, more in-depth analysis of the whip's dynamics is needed to identify relevant task execution priorities.

### 4.3. Rhythmicity in both discrete and rhythmic performance styles

Prior to comparing the two execution styles, we verified to what degree the instruction of rhythmicity was satisfied. The variability of trial durations in rhythmic performance was 6%, which is remarkably low and compared roughly to the variability reported for rhythmic tapping in synchrony with a metronome-prescribed frequency [38,39]. It is likely that this relatively invariant pattern was also dictated by the inertia of the whip that followed the control via the handle. Nevertheless, it may also be speculated that this robust cyclic pattern is the expression of a stable limit cycle behaviour, although this interpretation needs further verification via perturbation studies. Surprisingly, in the discrete style, the variability was also relatively low, only 12%. This repetitiveness is even more astonishing, as the inter-trial duration included a complete pause for the whip and the experimenter's go signal that was apparently timed randomly. Nevertheless, the fact that rhythmicity was established over the experimenter-participant dyad was noteworthy. Unintended rhythmicity over a sequence of similar actions has been reported before in a series of throwing actions that had the exclusive goal to hit a target [40,41]. Such rhythm ties the complex actions together and is another manifestation of how humans control the whip by simplifying its complex dynamics.

### 4.4. Discrete and rhythmic performance

Complementary to these findings were the differences and similarities in performance between the two styles. While it may still sometimes be assumed that insights into discrete movements generalize to rhythmic movements by simple concatenation, it became more than evident that, when handling the complex whip, continuous rhythmic execution is far from a concatenation of discrete movements. Several features underscored this realization. Performance was overall better in the discrete style, although the rhythmic style showed small improvements across blocks. Differences were also visible in how participants initiated the ‘throw’ by extending and aligning the whip with the target direction and how tip speed related to performance success. Although the hand speed trajectories showed a bimodal profile in both styles, the first peak arose from very different actions. The hand trajectory that prepared the throw to inject energy into the whip exhibited different peak speeds. Peak hand speed was higher in the discrete style and correlated with better accuracy, while it showed the opposite relation with error in the rhythmic style. In sum, analyses of discrete and rhythmic execution suggest distinct control strategies in the two styles. Despite these differences, the whip exhibited an unfolding pattern manifest in whip markers successively reaching peak speed. This pattern was present in both styles regardless of performance quality and emphasized the centrality of the whip.

### 4.5. Naturalistic movements studied in real environments

While simplifying movements to gain quantitative insight follows long-standing scientific tradition, it is not difficult to recognize that aspects of the fully fledged functional behaviour may be ‘controlled out’. For example, recent work on a throwing task compared performance in a real environment with a simplified matched virtual task and highlighted significantly different learning curves in the two environments [42]. Hence, insights into real throwing cannot be extrapolated from a simplified virtual task with its specificities. It is well recognized that human behaviour has all the known features of complex systems––high-dimensional, nonlinear, and hierarchical. In addition, continuous coupling with a high- or infinitely-dimensional object like the whip makes any methodological reduction even more problematic. Fortunately, advances in data recording and analysis have made it easier to address real-world behaviour although, evidently, challenges remain. This study aimed to capitalize on these advances to shed light on the coordinative strategies employed when manipulating a whip.

In motor neuroscience, only a small set of studies have examined natural behaviours with little or no reduction for scientific study. In the general area of locomotion, only a small set of studies left the treadmill and investigated walking overground including balancing and navigating on complex terrain [43–46]. To understand whole-body coordination, Haar and colleagues chose playing pool as an example task to characterize the multi-layered muscular and biomechanical variability [47]. A number of studies examined ball and dart throwing tasks and juggling in real settings and assessed the mapping between task-relevant variables and score-relevant parameters [48–53]. Though still comparatively few, these studies showed how research can progress toward understanding functional behaviour. The study presented here is another step in this direction.

### 4.6. Recording challenges of real three-dimensional whip motion

To our knowledge, the only experimental studies on whip behaviour to date have focused on its supersonic crack, using techniques such as stroboscopic photography or video shadowgraphy to visualize air pressure changes [54–57]. While the physics of whip cracking has been studied in simplified two-dimensional models with complementary recordings [36,54,56,58], no research to date has recorded or quantified three-dimensional whip movement. Both the extreme speed, flexion and torsion of the whip, and its non-isotropic composition and dynamics due to the thinner end pieces, fall and cracker, have made motion capture prohibitive. Part of that challenge was resolved here by removing the fall and the cracker of the whip, which in turn reduced the speed of the tip to approximately 50 m s^−1^ (compared to six-fold greater values of the cracker's speed). Note that even current markerless motion capture is not yet able to render reliable recordings of such high-speed motion. For the present research, reflective markers were developed that were concentrically threaded around the whip and thereby rendered 98% of the whip data available. Previous recording with conventional markers had lost 40% of markers, especially those closer to the tip of the whip [26]. In addition, the target was designed to be sufficiently sturdy and minimized tangling of the whip, while reliably recording contacts by the whip. These customizations yielded reliable kinematics of the fast-moving flexible object that allowed detailed analyses of the whip's configurations throughout the throwing action.

### 4.7. Inter-individual differences in task performance and improvement

The 16 participants in this experiment displayed a wide range of performance scores between 0 and 71% and errors varied between 3 cm and 35 cm per block. In their studies on naturalistic throwing, d'Avella and colleagues stressed that the significant inter-individual differences in unconstrained behaviour should not be averaged over, but rather regarded as a source of insight [59,60]. A different line of research showed that in naturalistic catching individual strategies emerged that resulted in different performance levels [61,62]. Recent work of the same group also extended a variability decomposition method, previously developed by Sternad and colleagues, to higher dimensional realistic behaviour to demonstrate how variability is shaped in the solution spaces of these redundant systems [63]. Note that manipulating a whip shares qualitative similarity with throwing as the moment of peak hand speed injects energy into the whip. Hence, peak hand speed was selected as corresponding analogue to the release moment in throwing a ball or any other projectile [60,64,65]. This analogy also suggests that any experience in volleyball, baseball and tennis may have influenced participants' performance with the whip [65]. Hence, the broad performance range is also likely due to participants’ different athletic experiences in addition to general physical fitness and coordinative abilities.

It is also notable that changes across practice blocks remained very limited, indicating that any improvements in this demanding skill take much longer than a single 1-h-long recording session. For comparison, a professional whip artist, Adam Winrich, performed a similar target-hitting task in our laboratory and showed approximately 90% success rate in both styles [26]. Indeed, whip artists with their amazing control over the whip's evolution have gone through many years of intensive practice to reach their exquisite skill. Hence, the improvements in rhythmic execution may be more adequately described as familiarization than true learning, although a reliable answer awaits further study.

### 4.8. Control strategies in humans and robots

What control strategies are employed in controlling a whip? Manipulation of flexible objects is still a well-recognized challenge in robotics [66]. The currently predominant approach invokes internal models of the object that the controller learns and uses for its control [67,68]. Although this approach has multiple lines of support in both human and robotic research, it may encounter problems when scaling up to this dimensionally vast spatio-temporal dynamics. Research on the manipulation of cable-like structures has proceeded by empirically matching control parameters to those observed in human behaviour [69–71]. As human control parameters are far from understood, a complementary simulation study of our group suggested a simpler control scheme. A multi-joint arm model manipulated a high degree-of-freedom whip model to hit a target located at a range of different locations. Importantly, the controller used a single submovement and constant joint mechanical impedances to control the arm joints and, agnostic about the whip dynamics, succeeded in hitting the target [72,73]. Despite these advances, a lot more research is needed to further advance control algorithms that come close to human dexterity.

## Data Availability

Data and relevant code for this research work are stored in GitHub: (https://github.com/dondestamos/WhipTask_PerformanceWhipHand) and have been archived within the Zenodo repository: http://doi.org/10.5281/zenodo.6987213 [74]. Additional data related to this paper may be requested from the authors. The data are provided in electronic supplementary material [75].
